# SVIM: structural variant identification using mapped long reads

**DOI:** 10.1093/bioinformatics/btz041

**Published:** 2019-01-21

**Authors:** David Heller, Martin Vingron

**Affiliations:** Department of Computational Molecular Biology, Max Planck Institute for Molecular Genetics, Berlin, Germany

## Abstract

**Motivation:**

Structural variants are defined as genomic variants larger than 50 bp. They have been shown to affect more bases in any given genome than single-nucleotide polymorphisms or small insertions and deletions. Additionally, they have great impact on human phenotype and diversity and have been linked to numerous diseases. Due to their size and association with repeats, they are difficult to detect by shotgun sequencing, especially when based on short reads. Long read, single-molecule sequencing technologies like those offered by Pacific Biosciences or Oxford Nanopore Technologies produce reads with a length of several thousand base pairs. Despite the higher error rate and sequencing cost, long-read sequencing offers many advantages for the detection of structural variants. Yet, available software tools still do not fully exploit the possibilities.

**Results:**

We present SVIM, a tool for the sensitive detection and precise characterization of structural variants from long-read data. SVIM consists of three components for the collection, clustering and combination of structural variant signatures from read alignments. It discriminates five different variant classes including similar types, such as tandem and interspersed duplications and novel element insertions. SVIM is unique in its capability of extracting both the genomic origin and destination of duplications. It compares favorably with existing tools in evaluations on simulated data and real datasets from Pacific Biosciences and Nanopore sequencing machines.

**Availability and implementation:**

The source code and executables of SVIM are available on Github: github.com/eldariont/svim. SVIM has been implemented in Python 3 and published on bioconda and the Python Package Index.

**Supplementary information:**

Supplementary data are available at *Bioinformatics* online.

## 1 Introduction

A typical human genome differs from the reference genome at ∼4–5 million sites amounting to ∼20 million altered bases (1000 Genomes Project Consortium, 2015). These variations can be categorized into single-nucleotide polymorphisms (SNPs), small insertions and deletions (Indels) and structural variation (SV) affecting a larger number of base pairs. Typically, differences larger than 50 bp are considered SVs although definitions vary and sometimes overlap with those of Indels.

Studies have shown that in human more base pairs are altered due to SV than due to SNPs (Redon *et al.*, 2006; Weischenfeldt *et al.*, 2013). Additionally, SVs are enriched 50-fold for expression quantitative trait loci when compared to SNPs (Sudmant *et al.*, 2015). Unsurprisingly, SVs have a major influence on human diversity and are implicated in a wide range of diseases from autism and other neurological diseases to cancer and obesity (Sebat *et al.*, 2007; Weischenfeldt *et al.*, 2013). Consequently, the characterization of SVs is of major importance to human medicine and genetics alike. It can contribute to the early detection of disorders and can help to elucidate their underlying genetic and molecular processes (Gonzalez-Garay, 2014). In other organisms such as plants, SVs play an equally important role by driving phenotypic variation and adaptation to different environments (Saxena *et al.*, 2014).

**Fig. 1.**
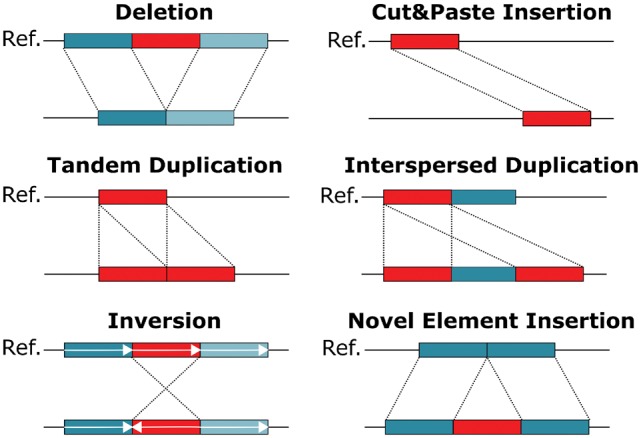
Schematic overview of different SV classes. SVs can be categorized into deletions, cut&paste insertions, tandem and interspersed duplications, inversions and novel element insertions. Each SV class is depicted in an individual genome (lower line) when compared to the reference genome (upper line). The region being rearranged is marked in red

Next generation sequencing has enabled the identification of SNPs and small Indels to a high resolution. SVs, however, are much harder to detect. One reason is that SVs encompass a diverse range of modifications. While SNPs are simple base pair substitutions, the term ‘SV’ summarizes many different phenomena. Typically, different classes of SVs are distinguished, such as deletions, inversions and insertions. Definitions for some of these classes vary in the literature. For the purpose of this work, we define six different SV classes which are visualized in Figure 1: deletions, cut&paste insertions, tandem and interspersed duplications, inversions and novel element insertions. The main drivers behind interspersed duplications in human are mobile element insertions, such as Alu, LINE1 and SVA elements. They duplicate using retrotransposition and in total represent ∼25% of all human SV (Stewart *et al.*, 2011; Sudmant *et al.*, 2015). DNA transposons, although now inactive in mammals (excepts bats) are active in plants and lower-order animals (Huang *et al.*, 2012). They use a cut&paste mechanism to move in the genome and therefore motivated the inclusion of cut&paste insertions as a separate SV class.

There exists a wide variety of tools for SV calling from short reads (Pabinger *et al.*, 2014) but despite ongoing efforts, the discovery of SVs from short-read data remains challenging (English *et al.*, 2015). Studies have estimated that short-read methods suffer from poor sensitivity down to 10% particularly for small SVs shorter than 1 kbp (Chaisson *et al.*, 2015; Huddleston *et al.*, 2016). In contrast to SNPs where discovery and sequence resolution can be performed simultaneously, SVs are discovered mainly indirectly using short paired-end reads. Their alignments are examined for characteristic signatures, such as inconsistently mapping read pairs, split reads and changes in read depth (Alkan *et al.*, 2011). These signatures can only be indirect evidence in favor of certain SV classes but are unable to fully characterize the SV. The main limitation here is that most SVs are simply larger than the short reads. The accurate detection of SVs is, besides their diversity, hampered by their association with repeat regions, biases in the sequencing technology and the additional complexity of diploidy (Carvalho and Lupski, 2016; Huddleston and Eichler, 2016; Willems *et al.*, 2014).

To characterize the full spectrum of human genetic variation, long-read sequencing technologies that generate reads with an average length of tens of kilobases show many advantages. The long reads can be mapped with greater accuracy which enables the sequencing of repetitive and low-complexity regions (Chaisson and Tesler, 2012; Loomis *et al.*, 2013). Unlike with short reads, SVs are often spanned by a single long read. This enables the direct detection and full characterization of the SVs. Consequently, several studies confirmed that a substantial number of SVs that are missed by short-read approaches can be identified with long reads (English *et al.*, 2015; Huddleston *et al.*, 2016; Merker *et al.*, 2018). Two commercial long-read sequencing solutions exist to date: single-molecule real-time (SMRT) sequencing by Pacific Biosciences (PacBio) and Nanopore sequencing by Oxford Nanopore Technologies (ONT). Both technologies have the same drawbacks: high error rates of ∼5–15% with dominating Indel errors and still high costs compared to short-read sequencing.

Similarly to the detection of SVs from short-read data, the first step toward SV detection from long reads is often the alignment of the reads to a reference genome. Depending on the alignment tool used to produce the alignments, SV detection results can vary substantially as Sedlazeck *et al.* showed for their tool *Sniffles* (Sedlazeck *et al.*, 2018a). In that study, SV-spanning long reads were aligned with seven different aligners. Their results showed that one particular aligner, *NGMLR*, outperformed all the others (including *BWA-MEM*, *Minimap2*, *LAST* and *BLASR*) on the task (Sedlazeck *et al.*, 2018a). In our study, we analyzed read alignments by *NGMLR* to detect SVs. In the Supplementary Material, however, we include results for *Minimap2* which is an order of magnitude faster than *NGMLR* (Li, 2018).

Read alignments alone are not sufficient to detect and characterize SVs. Dedicated SV callers are needed to collect and interpret evidence from the read alignments. Recently, three methods have been developed for calling SVs based on long reads (Sedlazeck *et al.*, 2018b). *PBHoney* and *SMRT-SV* are designed specifically for PacBio reads while *Sniffles* supports PacBio and ONT reads (English *et al.*, 2014; Huddleston *et al.*, 2016; Sedlazeck *et al.*, 2018a).

*PBHoney* comprises two different variant identification approaches (English *et al.*, 2014). The first approach, *PBHoney-Spots*, exploits the stochastic nature of the errors in PacBio reads. It scans read alignments (usually produced by the read aligner *BLASR*) and recognizes SVs by an increase in error and a subsequent decrease in error along the reference sequence. The second approach, *PBHoney-Tails*, analyzes the soft-clipped (i.e. unmapped) read tails from a *BLASR* alignment. It extracts such tails from the BLASR output and realigns them to the reference. Then, SVs are detected by clustering the resulting piece-alignments based on their location and orientation.

*SMRT-SV* scans PacBio alignments for SV signatures, such as spanned deletions, spanned insertions and soft-clipped read tails (Huddleston *et al.*, 2016). Clusters of such events are validated with a local *de-novo* assembly of the reads overlapping the locus and subsequent alignment of the assembly to the reference.

*Sniffles* uses signatures from split-read alignments, high-mismatch regions and coverage analysis to identify SVs (Sedlazeck *et al.*, 2018a). To overcome the high error rate in the reads, it evaluates candidate SVs based on features such as their size, position and breakpoint consistency.

All three methods regard SV (i.e. deletions, insertions, inversions) as rearrangements occurring in a single genomic locus. However, SV often involves multiple genomic loci, such as for a mobile element which is reverse-transcribed from a source region and inserted at another location. The higher read lengths of PacBio and ONT reads allow to link both loci much more efficiently and confidently than was possible with short paired-end reads. Nevertheless, existing methods ignore this type of information and are only able to detect the isolated destination location of the mobile element insertion.

In this study, we introduce SVIM, a computational method for the sensitive detection and accurate classification of five different classes of SVs from long-read sequencing data. We describe the three core components of the approach and our methodology for evaluation on simulated and real datasets. Our results demonstrate that SVIM reaches substantially higher recall and precision than existing tools for SV detection from long reads. Unlike other methods, SVIM has been specifically designed to distinguish three separate classes of large insertions: interspersed duplications, tandem duplications and insertions of novel elements. To our knowledge, it is the only tool capable of identifying not only the insertion location of an interspersed duplication but also its potential genomic origin using long reads. We demonstrate this capability on a small number of high-scoring interspersed duplications identified in the NA12878 individual. Furthermore, we compare SV callsets produced by SVIM on reads from PacBio and Nanopore data. Finally, we compare the runtimes of different SV callers including SVIM.

## 2 Materials and methods

**Fig. 2.**
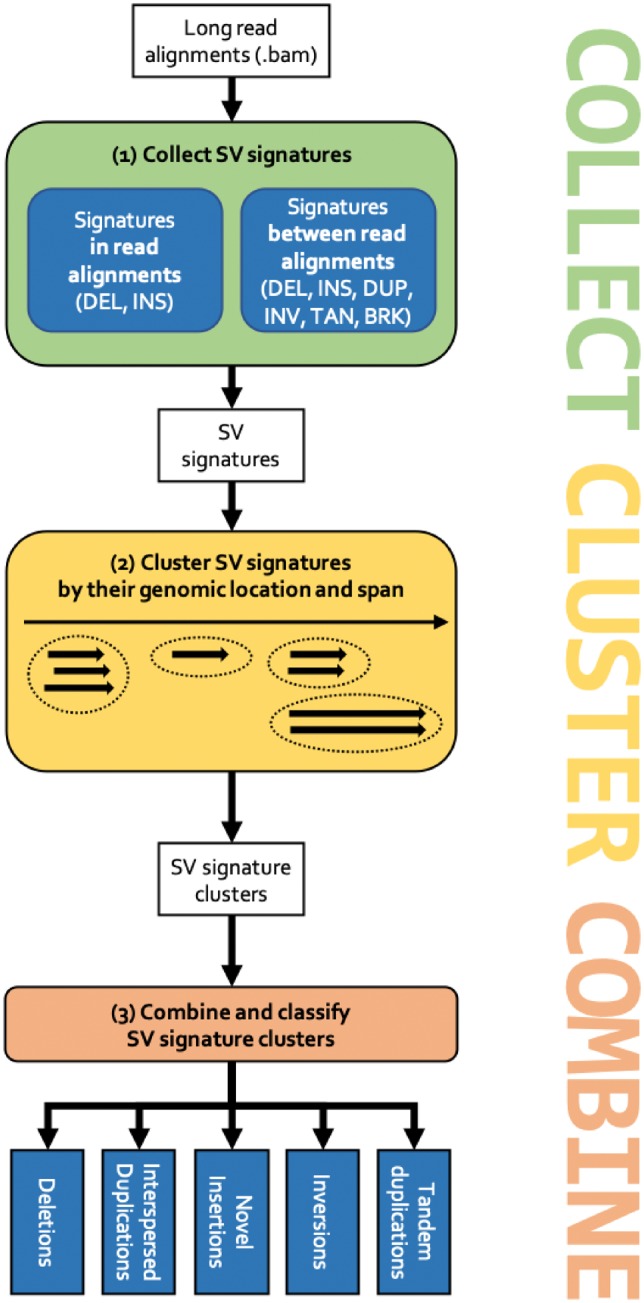
The SVIM workflow. (1) Signatures for SVs are collected from the input read alignments. SVIM collects them from within alignments (intra-alignment signatures) and between alignments (inter-alignment signatures). (2) Collected signatures are clustered based on their genomic position and span. (3) Signature clusters from different parts of the genome are combined to distinguish five different classes of SVs: deletions, interspersed duplications, novel insertions, inversions and tandem duplications

SVIM implements a pipeline of three consecutive components (see Fig. 2). First, SV signatures are collected from each individual read in the input Sequence Alignment Map (SAM)/Binary Alignment Map (BAM) file (COLLECT). Secondly, the detected signatures are clustered using a graph-based clustering approach and a novel distance metric for SV signatures (CLUSTER). Thirdly and lastly, multiple SV events are merged and classified into higher-order events (i.e. events involving multiple regions in the genome) such as duplications (COMBINE). The three components are explained in the following.

### 2.1 Collection of SV signatures from individual reads

SVIM analyzes read alignments in SAM/BAM format (Li *et al.*, 2009) from a read aligner. Modern aligners, such as *NGMLR* and minimap2, try to find good linear alignments of entire reads. Nevertheless, they will split a chimeric read if its different segments can be better aligned separately. Due to these split alignments, the SAM/BAM output from these aligners can contain multiple alignments for each read (one for each aligned read segment). SVIM extracts signatures for SVs from the SAM/BAM file by analyzing one read at a time. We define SV signatures as discordant alignments of a read that point to the presence of one or several possible SVs in the sequenced genome. SVIM searches for two types of signatures:

*Intra-alignment signatures* are large alignment gaps in the reference or in the read. They can be found in the CIGAR strings of individual SAM/BAM entries.

*Inter-alignment signatures* are discordant relative alignment positions and orientations of a read’s alignment segments. To illustrate this type of evidence, imagine an inversion that is spanned by a single read. The aligner will split the read into three alignment segments: one segment upstream of the inversion, another segment for the inverted region (INV), and a third segment downstream of the inversion. Due to the inversion, the middle segment will have a different mapping orientation than the other two pieces. This and other types of inter-alignment signatures are detected by SVIM in a heuristic fashion.

This analysis yields six different types of SV signatures: (i) deleted regions (DEL), (ii) inserted regions (INS), (iii) INSs with detected region of origin (DUP), (iv) INVs, (v) tandem duplicated regions (TAN) and (vi) translocation breakpoints (BRK). Some of these evidence types (e.g. INVs) indicate one particular SV class. Others could indicate several possible SV classes. An INS, for instance, can indicate both a duplication or a novel element insertion.

### 2.2 Clustering of SV signatures

The collection of signatures from the alignments is only the first step to accurately detect SVs. Subsequently, signatures from multiple reads need to be merged and criteria have to be found to distinguish correct signatures from multiple types of error artifacts (e.g. sequencing error, alignment error). To achieve this, we combine a graph-based clustering approach with a novel distance metric for SV signatures. The aim is to merge signatures of the same SV even if their positions vary slightly due to sequencing or alignment errors. At the same time, signatures from separate SVs need to be kept separate even if the two SVs lie close to each other.

The collected SV signatures can be viewed as quadruples $S_{i}=(T_{i},C_{i},B_{i},E_{i})$ where *T* is one of the six different signature types defined above, *C* is the chromosome and *B* and *E* are the genomic start (begin) and end positions. One of the few distance metrics defined for such genomic intervals is the Gowda–Diday distance (Gowda and Diday, 1991). It combines (i) the distance between two intervals, (ii) their span difference and (c) their degree of overlap into a single numeric distance value. In our type of data (i.e. long-read alignments), however, we often observe little to no overlap between signatures originating from the same SV but from different long reads (see Supplementary Fig. S1). Nevertheless, signatures from the same SV often possess similar positions and spans.

Therefore, we introduce *span-position distance* as a novel distance metric for SV signatures. For two SV signatures *S*_1_ and *S*_2_, the span-position distance SPD consists of two components SD and PD: $\text{SPD}=\text{SD}(S_{1},S_{2})+\frac{\text{PD}(S_{1},S_{2})}{N}$. SD is the difference in span between both signatures [normalized to [0,1)] and is defined as $\frac{\left|(E_{1}-B_{1})-(E_{2}-B_{2})\right|}{\max(E_{1}-B_{1},E_{2}-B_{2})}$. PD is the difference in position between both signatures and is defined as $\min(|B_{1}-B_{2}|,|E_{1}-E_{2}|,|\frac{B_{1}+E_{1}}{2}-\frac{B_{2}+E_{2}}{2}|)$. *N* is a user-defined normalization constant which regulates the relative importance of SD and PD. In our analyses, setting *N *=* *900 returned the best results. Intuitively, this setting means that two signatures that are 900 bp apart (PD = 900) but have the same span (SD = 0) would have the same SPD as two signatures with extremely different spans (SD≈1) but the same position (PD = 0).

To perform clustering, we follow a graph-based approach similar to the one used by the variant finder *CLEVER* (Marschall *et al.*, 2012). Initially, we transform the set of collected SV signatures into an undirected graph. While *CLEVER* identifies nodes with alignments of short paired-end reads, each node in our graph represents an SV signature. We draw an edge between two nodes (i.e. signatures) if the span-position distance between the two signatures is smaller than a user-defined threshold *T*. Systematic evaluation of different settings for this parameter yielded *T *=* *0.7 as an optimal setting for our simulated human datasets (data not shown). An edge between two nodes expresses our confidence that the two signatures represented by the nodes express the same SV allele. From the graph, we produce signature clusters by extracting maximal cliques with an efficient implementation of the Bron–Kerbosch algorithm (Bron and Kerbosch, 1973; Hagberg *et al.*, 2008). As a consequence, each signature cluster is a maximal group of SV signatures that can be jointly assumed to express the same SV in the donor genome.

Finally, SVIM computes a score for each cluster based on four features:

1. The number n∈(0,40] of signatures in the cluster where at most 20 of each class (intra-alignment or inter-alignment) are taken into account.
2. An additional bonus b∈[0,30] for the existence of at least one signature from each of the two classes. One or more intra-alignment signatures earn a bonus of 10 while one or more inter-alignment signatures earn an additional bonus of 20.
3. A score $s_{p}\in [0,10]$ based on the standard deviation *s*_pos_ of the genomic positions of the signatures in the cluster normalized by their average span. $s_{p}=10*(1-\min(1,s_{\text{pos}}/\bar{\mathrm{span}}))$
4. A score $s_{s}\in [0,20]$ based on the standard deviation *s*_span_ of the genomic spans of the signatures in the cluster normalized by their average span. $s_{s}=20*(1-\min(1,s_{\text{span}}/\bar{\mathrm{span}}))$

By summing up these four components we obtain a score S∈(0,100] to discern trustworthy signature clusters from artifacts, such as sequencing or alignment artifacts. Trustworthy events are characterized by many intra- and inter-alignment signatures that exhibit high concordance regarding their genomic position and span.

### 2.3 Combination and classification of SVs into five SV classes

**Fig. 3.**
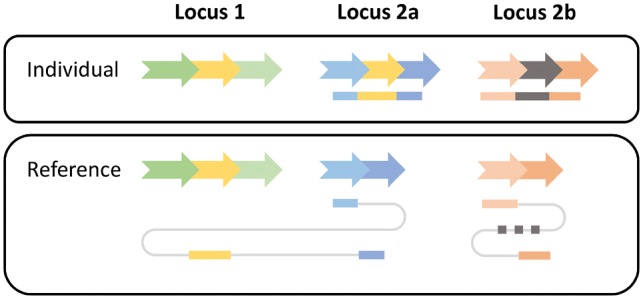
Read signatures for an interspersed duplication and a novel element insertion. A genomic segment (yellow arrow) has been copied from locus 1 to locus 2a in an individual genome. Additionally, a novel genomic segment (gray arrow) has been inserted in locus 2b. Two reads are generated from the individual (top) and mapped to the reference genome (bottom). The first read (blue-yellow) consists of three segments. They are mapped individually to the reference genome. The two blue segments are mapped to locus 2a exhibiting an insertion signature. The yellow segment is mapped to locus 1 indicating the origin of the insertion. The second read (orange-gray) exhibits a similar insertion signature at locus 2b but as the inserted gray segment is unmapped its origin cannot be determined

The third component in the workflow analyzes and combines the SV signature clusters to classify events into five SV classes: deletions, inversions, novel element insertions, tandem duplicaitons and interspersed duplications. Because the confident distinction of interspersed duplications and cut&paste insertions solely based on sequencing reads is impossible, we classify both as interspersed duplications. Nevertheless, we annotate duplications where the region of origin seems to be deleted in the sequenced individual (i.e. a deletion overlaps the genomic origin) as potential cut&paste insertions. While INV, DEL and TAN signature clusters can be directly reported as inversions, deletions and tandem duplications, respectively, the other three signature classes (INS, DUP and BRK) are more complex. The reason is that interspersed duplications are not characterized by only one genomic region but two—a genomic origin and a genomic destination. To capture and classify these higher-order events, SVIM needs to combine multiple signature clusters and therefore makes the following distinctions (see also Fig. 3):

- DUP signature clusters are called as interspersed duplications. If the genomic origin overlaps a deletion call, the duplication is marked as potential cut&paste insertion.
- INS signature clusters that are close to matching BRK are called as interspersed duplications. If the genomic origin (as defined by the BRK) overlaps a deletion call, the duplication is marked as potential cut&paste insertion.
- The remaining INS signature clusters are called as novel element insertions.

### 2.4 Implementation and usage

SVIM has been implemented in Python and is available at github.com/eldariont/svim. It can be easily installed via bioconda or the Python Package Index. As input, SVIM expects either raw reads (in FASTA or FASTQ format) and a reference genome (in FASTA format) or already aligned reads in BAM format. It outputs detected SVs in five separate BED files (one for deletions, interspersed and tandem duplications, inversions and novel insertions, respectively). Additionally, a VCF file with all SV results is produced.

### 2.5 Evaluation methodology

In this study, we compared our tool, SVIM (v0.4.1), to three other SV detection methods: *PBHoney-Spots*, *PBHoney-Tails* (both PBSuite v15.8.24) and *Sniffles* (v1.0.8). All three tools are designed for the application on long-read sequencing data. For *Sniffles* and SVIM, reads were aligned with *NGMLR* (v0.2.7) or *minimap2* (v2.12-r836-dirty). For *PBHoney*, reads were aligned with *BLASR* (v5.3.4323a52). We did not compare against short-read SV callers because they have been shown to exhibit lower recall than methods relying on long reads (Chaisson *et al.*, 2015; Huddleston *et al.*, 2016; Sedlazeck *et al.*, 2018a). We also did not compare against *SMRT-SV* because it is not a stand-alone tool but a software pipeline applying several alignment, detection and assembly steps with various other tools. It detects only three SV classes and is computationally more demanding than pure alignment-based tools.

We evaluated all tools on two types of data. Firstly, we generated a simulated genome from which we sampled *in-silico* PacBio sequencing reads with known SVs. This provided us with a complete set of fully characterized SVs for evaluation. Secondly, we used publicly available sequencing reads from PacBio and Nanopore sequencers. We compared the precision and recall of the three methods. Precision is defined as the fraction of detected SVs that are correct (requiring 50% reciprocal overlap between detected and correct SVs). Recall is defined as the fraction of correct SVs that have been detected (with 50% reciprocal overlap). Results for a more lenient and a more stringent overlap requirement of 1 and 90%, respectively, can be found in the Supplementary Material. Both precision and recall require a suitable gold standard set of high-confidence SVs for the given genome (i.e. a set of correct SVs).

**Fig. 4.**
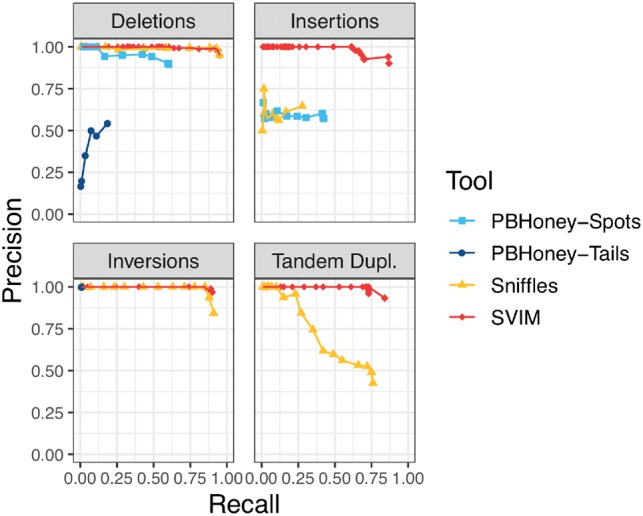
Comparison of SV detection performance on a 6× coverage homozygous simulated dataset. SVIM consistently yielded better recall (*x*-axis) and precision (*y*-axis) than the other tools for the recovery of INSs and tandem duplications. For the recovery of deletions and inversions, *Sniffles* reached the same recall as SVIM. The different points for each tool represent multiple settings of the tools’ most important parameters (see Section 2.5). *PBHoney-Spots* only detects deletions and INSs and *PBHoney-Tails* does not detect duplications. Recall and precision were calculated using a required reciprocal overlap of 50% between variant calls and the original simulated variants

**Fig. 5.**
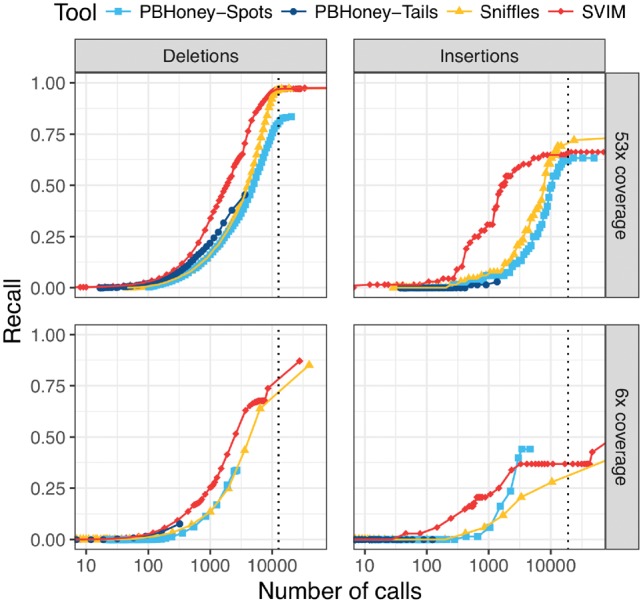
Comparison of recall on a 53× coverage public PacBio dataset and a 6× coverage subset with 2676 high-confidence deletion and 68 insertion calls. For each tool and different thresholds, the number of SV calls with score above the threshold (log-scale) is plotted against the recall. The upper and lower panels show performance on the full dataset and a randomly sampled 6× coverage subset of the data, respectively. SVIM reached the same recall with fewer calls than other tools. The vertical dotted lines denote the average number of deletions and insertions to expect in an individual as recently reported using a *de-novo* assembly approach (Chaisson *et al.*, 2018). Recall was calculated using a required reciprocal overlap of 50% (deletion calls) and 1% (insertion calls), respectively, between variant calls and the gold standard variants

**Fig. 6.**
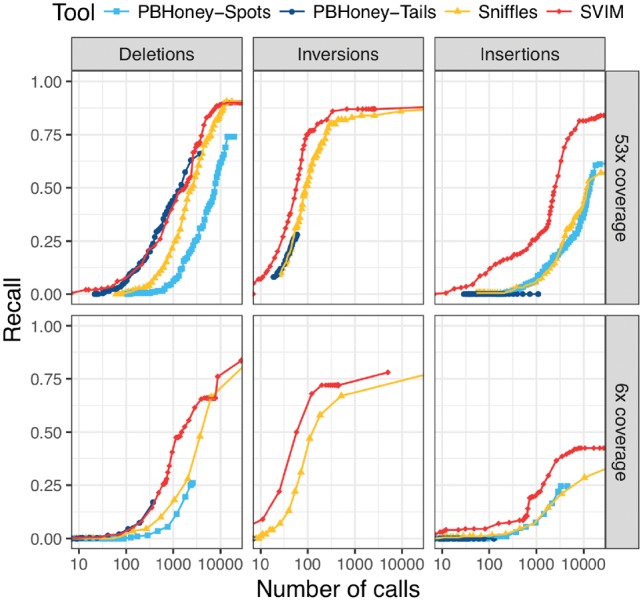
Comparison of recall from NA12878 reads aligned to an altered reference genome. For each tool and different thresholds, the number of SV calls with score above the threshold (log-scale) is plotted against the recall. The upper and lower panels show performance on the full dataset and a randomly sampled 6× coverage subset of the data, respectively. In all six panels, SVIM outperformed all the other tools and reached substantially higher recall for similar numbers of calls. The improvement was most prominent for insertions. Recall was calculated using a required reciprocal overlap of 50% between variant calls and the original implanted variants

As expected, recall and precision reached by the different tools depend heavily on tool parameters, particularly score or support thresholds. More relaxed thresholds (i.e. yielding more SVs) increase recall but decrease precision while stricter cutoffs achieve the opposite. Consequently, we ran all four tools with different settings of their most important parameter: For SVIM we applied different score cutoffs (0–100). *Sniffles* was run with different settings of the *min_support* parameter (1–60). For *PBHoney-Spots*, we varied the *minErrReads* parameter and for *PBHoney-Tails* we varied the *minBreads* parameter (both 1–60). We visualized the performance of the tools by plotting each parameter setting as a distinct point in Figures 4–6. Besides that one parameter, we used the default settings for all other tool parameters except PBHoney Spots’ *spanMax* parameter which we set to 100 000 (100 kb).

#### 2.5.1 Simulated data

We simulated 600 homozygous SVs by altering the sequence of chromosomes 21 and 22 in the hg19 reference genome. More precisely, we implanted 200 deletions, 100 inversions, 100 tandem duplications and 200 interspersed duplications with the R package *RSVSim* (Bartenhagen and Dugas, 2013). The package estimates the distribution of SV sizes from real datasets and simulates the association of SVs to various kinds of repeats. The resulting genome contained SVs between 50 bp and 10 kbp in size. Subsequently, reads were simulated from this genome to generate 10 different datasets with coverages between 6 and 60× with the tool *SimLoRD* (Stöcker *et al.*, 2016). *SimLoRD* imitates the error model of SMRT reads to simulate realistic PacBio reads.

To simulate heterozygous SVs, we adapted the previously described approach only slightly. Instead of sampling all reads from the altered reference genome, half of the reads were sampled from the original reference genome. Consequently, reads from the original (wild-type) reference genome and the altered genome each amounted to 50% of the total coverage.

The comparison between different tools was complicated by the fact that each tool is designed to detect different SV classes. *PBHoney* is able to detect deletions, INSs, inversions and BRKs. *Sniffles* is additionally capable of identifying tandem duplications and complex events. Because only SVIM distinguishes between duplications and novel element insertions, we compared the tools on four common basic SV classes in the simulated datasets: deletions, INSs (i.e. inserted sequence from duplications and novel element insertions), inversions and tandem duplications. Because *Sniffles* tends to call intra-chromosomal duplications as very large deletions or inversions (see github.com/fritzsedlazeck/Sniffles/issues/23), we omitted deletion and inversion calls by *Sniffles* that were larger than 100 kbp to ensure a fair comparison. To obtain calls of INSs from SVIM, we use the union of its interspersed duplication and novel element insertion calls.

#### 2.5.2 Real data

Simulation cannot reflect all aspects of biological data. Therefore, we used real PacBio and Nanopore data for the second part of our analysis. This part consisted of three separate experiments. For the first two, we utilized a real 53× coverage dataset of the NA12878 individual from a PacBio RS II machine (Genome in a Bottle consortium; Accession SRR3197748) (Zook *et al.*, 2014). To assess the influence of sequencing coverage on SV detection performance, we produced a corresponding low-coverage subset of the dataset by sampling reads randomly to 6× coverage. With these two PacBio datasets, we performed two separate analyses. Firstly, we evaluated our method with a published benchmark sample of 2676 high-confidence deletions and 68 high-confidence insertions (Parikh *et al.*, 2016). Secondly, we implanted SVs into the reference genome and aligned the PacBio reads to this altered reference. Implanting an SV into the reference genome causes the original reads to contain the inverse of the SV that was implanted. With this approach, three types of SVs were simulated: (i) 200 deletions were simulated by inserting sequence into the reference genome; (ii) 100 inversions were simulated by inverting regions in the reference and (iii) 200 insertions were simulated by deleting regions in the reference. Unfortunately, duplications could not be simulated because this would have required the identification and alteration of existing duplications in the reference genome.

In a third experiment, we compared the 53× coverage PacBio dataset of the NA12878 individual with a 26× coverage Nanopore dataset of the same individual (Jain *et al.*, 2018, release 5). We evaluated our method with the high-confidence callset described above and analyzed the overlap between the three callsets (PacBio, Nanopore and high-confidence callset).

The NA12878 datasets are more realistic than the simulated dataset but impose the limitation that there exists no complete gold standard set of SVs. As a consequence of using an incomplete gold standard for evaluation, precision could not be accurately measured. Putative ‘false positives’ could have been true but simply not contained in the incomplete gold standard. Therefore, we compared the tools only based on their recall in relation to the number of calls.

## 3 Results

### 3.1 Evaluation with simulated reads

As described in the Section 2, we implanted SVs from four different classes into a reference genome. Reads sampled from this synthetic genome were then analyzed with SVIM, *PBHoney-Tails*, *PBHoney-Spots* and *Sniffles*. Results for the 6× coverage homozygous dataset can be found in Figure 4. For a comparison of results across all coverages from 6 to 60× see Supplementary Figure S2.

Regardless of coverage, SVIM achieved substantially better results than all other tools in the recovery of INSs and tandem duplications. With 6× coverage and homozygous SVs, SVIM reached average precisions (AP) of 86% (INSs), and 83% (tandem duplications) for the two classes while the second best tools, *PBHoney-Spots* and *Sniffles* respectively, reached 25 and 54%. In the recovery of deletions and inversions, SVIM and *Sniffles* reached equal results with AP of 94% (deletions) and 90% (inversions), respectively. In our experiments, *PBHoney-Tails* performed very poorly across all settings. It did detect only very few INSs, suffered from very low recall for inversions and poor precision for deletions. All these trends remain true for higher coverages as well (see Supplementary Fig. S2).

The simulated heterozygous dataset yielded similar results to those of the homozygous dataset (see Supplementary Fig. S5). While all tools reached slightly lower precision and recall, SVIM still outperformed the others for INSs (AP = 68% for 6× coverage) and tandem duplications (AP = 76%). In the detection of deletions and inversions, however, *Sniffles* and SVIM reached nearly equal results (AP = 90% and AP = 87%, respectively).

We explored whether more lenient (1%) or stringent (90%) overlap requirements for the calls would change the results (see Supplementary Figs S3, S4, S6 and S7). As it turned out, the overlap requirement had little effect on *Sniffles* and SVIM. Only *PBHoney-Spots* produced substantially worse results for more stringent overlap requirements suggesting that the tool has trouble finding accurate SV breakpoints.

To measure the influence of the input read alignments on SV calling, we also compared results for two long-read aligners, *NGMLR* and *minimap2* (see Supplementary Figs S8 and S9). The results indicate that SVIM is relatively robust to the choice of the aligner but benefits slightly from the more accurate alignment of reads covering insertions and tandem duplications by *NGMLR*. *Sniffles*, however, reaches considerably higher recall for insertions when analyzing alignments by *minimap2* compared to *NGMLR*. Visual inspection of the alignments revealed a difference in the way that reads covering insertions are aligned. While *minimap2* expresses insertions mainly as long reference gaps in the CIGAR string, *NGMLR* tends to split reads at insertions. Because *Sniffles* does not call insertions of sequence existing somewhere else in the genome (i.e. interspersed duplications) from split alignments, it reaches higher recall with *minimap2*.

### 3.2 Evaluation with real reads and high-confidence calls

While simulated datasets enable the comprehensive comparison of tools in a controlled and precise manner, they cannot reflect the full complexity of real sequencing data. Therefore, we analyzed a publicly available 53× coverage dataset of a human individual from a PacBio RS II machine and a random 6× coverage subset (see Section 2). To evaluate the detection performance of our tool, we first used a published benchmark set of 2676 high-confidence deletions and 68 high-confidence insertions.

Among all tools, SVIM was the most consistent across the different settings (see Fig. 5). It recovered substantially more deletions from the high-confidence call set than the other tools with the same number of SV calls. To reach a recall of more than 50%, SVIM needed 1932/2577 calls (53×/6× coverage) while *Sniffles* needed 4320/6333 calls. *PBHoney-Spots* needed even 5062 calls (53× coverage) and *PBHoney-Tails* did not reach this level of recall at all. A recent study by the Human Genome Structural Variation Consortium (HGSVC) used a multi-platform *de-novo* assembly approach for SV detection and found an average of 12 680 deletions per individual (Chaisson *et al.*, 2018). When we select tool thresholds closest to this mark, SVIM, *Sniffles*, *PBHoney-Spots* and *PBHoney-Tails* recover 97, 97, 80 and 46% of the high-confidence deletions from the full coverage dataset, respectively. All tools miss high-confidence calls across the entire size range (50 bp–140 kb). But while the false negatives of the first three tools are evenly distributed across the size spectrum, *PBHoney-Tails* particularly misses small events. For instance, it misses all high-confidence calls smaller than 100 bp and 69% of calls between 100 and 500 bp but only 24% of calls between 500 bp and 1 kb.

Although the results for insertions need to be considered with greater caution due to the small size (*n* = 68) of the high-confidence call set, SVIM reached a higher recall than all other tools for small numbers of calls. When we again select tool thresholds closest to the estimate of 18 919 insertions per individual from the HGSVC study (Chaisson *et al.*, 2018), SVIM, *Sniffles*, *PBHoney-Spots* and *PBHoney-Tails* recover 66, 72, 62 and 3% of high-confidence insertions from the full coverage dataset, respectively. Again, all tools miss high-confidence calls across the entire size range of the callset (12–379 bp).

### 3.3 Evaluation with real reads and an altered reference genome

As described in the Section 2, we obtained another reliable gold standard set of SVs (deletions, inversions, insertions) by implanting SVs into the reference genome and aligning the PacBio reads (53 and 6× coverage) to this altered reference. We evaluated all combinations of the three SV types and the two coverages. SVIM outperformed the other tools (see Fig. 6) in all six of these settings. In the recovery of deletions and inversions, SVIM reached a substantially higher recall than *PBHoney*. It also needed fewer SV calls to reach similar recall than *Sniffles* while the difference decreased for higher recall. The most striking difference was observed for the detection of insertions. While SVIM reached a recall of 84 and 43% with 20 000 calls (53 and 6× coverage, respectively), *PBHoney-Spots* reached 61 and 25% and *Sniffles* detected only 57 and 29% with the same number of calls. For full coverage, SVIM needed 2480 calls to reach a recall of 50% while *Sniffles* and *PBHoney-Spots* needed both more than 10 000 calls.

### 3.4 Interspersed duplications in NA12878

SVIM’s ability to link the genomic origin and destination of an interspersed duplication can yield interesting insights into the dynamics of genomic rearrangements. Our analysis of the NA12878 PacBio dataset with SVIM identified 27 high-confidence interspersed duplications with a score >30 (Supplementary Table S1). The genomic origin of 19 of them overlapped annotated retrotransposons. Among those, 10 and 2 represented complete and incomplete Alu insertions, respectively; 2 and 2 represented insertions of complete and incomplete LINE1 elements, respectively; 2 represented complete SVA elements and another one represented human endogenous retrovirus HERVK14. Strikingly, six duplications occurred from regions of the genome without annotated repeat elements indicating other formation mechanisms. Finally, we observed two duplications in the untranslated regions of three genes, BAZ2A, RBMS2 and PCMTD1.

### 3.5 Comparison of PacBio and Nanopore sequencing data

**Fig. 7.**
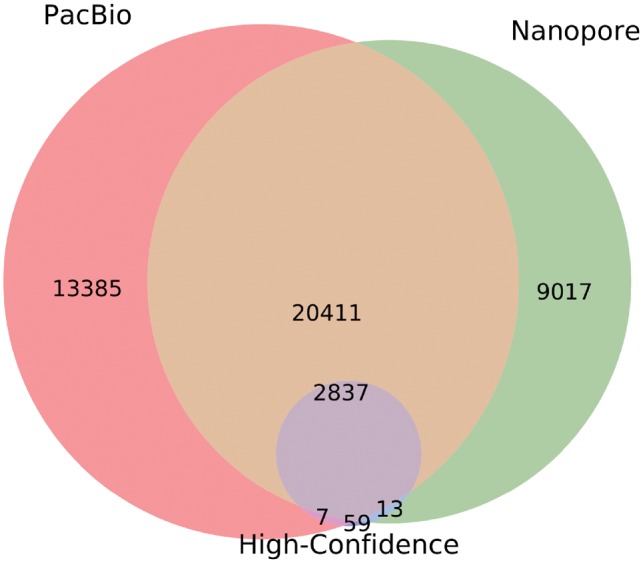
Venn diagram of three SV callsets for NA12878: SVIM calls on a 53× coverage PacBio dataset, SVIM calls on a 26× coverage Nanopore dataset and high-confidence calls from Parikh *et al.* (2016). Callsets were produced by merging SVIM calls with a score ≥40 for deletions, interspersed duplications and novel element insertions. Subsequently, the diagram was generated using *pybedtools* (Dale *et al.*, 2011) and *matplotlib_venn*

SVIM can detect SVs from both PacBio and Nanopore data. An evaluation with real reads and high-confidence calls demonstrated that SVIM’s performance on a 26× coverage Nanopore dataset is comparable to its performance on the 53× coverage PacBio dataset (see Supplementary Fig. S14). When we compared both SVIM callsets with the high-confidence callset, we found that all three callsets together yielded a total of 45 729 SVs (score cutoff of 40; see Fig. 7). A total of 22 461 or 49% of the calls were unique to one of the callsets with 13 ** **385 and 9017 SVs detected exclusively from the PacBio and Nanopore reads, respectively. However, 23 248 or 51% of the calls were made on both PacBio and Nanopore reads. It is reassuring that the vast majority (97%) of high-confidence calls were detected by both sequencing technologies.

PacBio and Nanopore sequencing exhibit similar error rates but slightly different error modes. While PacBio produces more insertion than deletion errors (Ross *et al.*, 2013), Nanopore shows the opposite tendency (Jain *et al.*, 2015). In concordance to these biases, SVIM detected 17 292 deletions from the Nanopore reads but only 12 782 deletions from the PacBio data (see Supplementary Fig. S15). Conversely, it detected 23 858 insertions from PacBio but only 14 986 insertions from Nanopore data (see Supplementary Fig. S16). Consequently, the majority of PacBio-only calls were insertions (90%) and the majority of Nanopore-only calls were deletions (65%). We could confirm the finding by Sedlazeck *et al.* that a large fraction (80%) of Nanopore-only calls lay in simple tandem repeats in contrast to only 35% of Pacbio-only calls (Sedlazeck *et al.*, 2018a).

### 3.6 Runtime evaluation

We compared the runtimes of *PBHoney-Spots*, *PBHoney-Tails*, *Sniffles* and SVIM on the same NA12878 dataset (53× coverage). *Sniffles* and SVIM were given input alignments produced by *NGMLR* while *PBHoney-Spots* and *PBHoney-Tails* were given *BLASR* alignments. The runtime was measured on an AMD EPYC 7601 (128 cores, 2.7 GHz, 1 TB memory). Only the runtime of SV detection was measured, excluding the time required for producing the alignments. All four tools analyzed the entire dataset in under 3 h (see Table 1). *PBHoney-Tails*, *Sniffles* and SVIM use only a single core and took 57 160, and 156 min, respectively. *PBHoney-Spots* is the only tool benefiting from multiple cores and took 145 min on 4 cores (608 min on only 1 core).

**Table 1.** Runtime comparison on the 53× coverage NA12878 PacBio dataset

Tool	Threads	CPU time (min)	Wall clock time (min)
PBHoney-Spots	1	601	608
PBHoney-Spots	2	561	284
PBHoney-Spots	4	558	145
PBHoney-Tails	1	56	57
Sniffles	1	159	160
SVIM	1	155	156

*Note*: Only the runtime of each tool is measured excluding the prior read alignment.

## 4 Discussion

SV is, besides single-nucleotide variation and small Indels, one of the main classes of genetic variation. The influence of SVs on human phenotype and disease makes them an important research target but their unique properties complicate their detection and characterization. Particularly SV detection methods using short-read technology suffer from low sensitivity. Long-read sequencing technologies such as PacBio SMRT sequencing and ONT Nanopore sequencing have the potential to alleviate these problems. In this study, we introduced the novel SV detection method SVIM. It employs a three-step pipeline to collect, cluster and combine SV signatures from long reads.

A comparison of SVIM with three competing tools on simulated and real data demonstrated that our method combines high sensitivity with high precision. Across all tools, deletions were the easiest to detect. Consequently, Sniffles and SVIM reached almost perfect precision and recall on the simulated data. On the real datasets, both tools still reached a recall of over 90% when setting thresholds using the HGSVC estimate of 12 680 deletions per individual (Chaisson *et al.*, 2018). This level of recall was maintained regardless of sequencing technology and evaluation method (high-confidence callset or altered reference). SVIM generally required fewer calls to reach the same recall as the other tools indicating that the best-scoring SVIM calls are more enriched in true variants than the other tools’ callsets of similar size. For the identification of inversions, *Sniffles* and SVIM exhibited equally strong performance although SVIM showed a slightly higher recall in the evaluation with an altered reference. It needs to be noted, however, that the evaluation of inversions had to rely fully on simulation due to the lack of a suitable gold standard set.

Differences between SVIM and the other tools were most prominent for INSs (i.e. interspersed duplications and novel element insertions). Across all simulations and real data evaluations, SVIM outperformed the other tools by a wide margin. The difference to *Sniffles* can be largely explained by their approach of analyzing split alignments. From such alignments, *Sniffles* only calls insertions of novel elements but does not detect insertions of sequence existing somewhere else in the genome (e.g. from mobile elements). The detection performance of tandem duplications could only be evaluated in the simulated dataset again due to the lack of a gold standard. What we observed is a big difference in precision between SVIM and *Sniffles* due to a large number of erroneous duplication calls by Sniffles.

What sets SVIM apart from existing SV callers is not only its improved detection performance but also its ability to distinguish three different classes of insertions purely based on alignment information. SVIM enables researchers to determine whether an insertion event is due to a tandem duplication, an interspersed duplication or the insertion of a novel element. Moreover, SVIM identifies the genomic origin of duplications which facilitates their functional annotation, e.g. into different classes of mobile elements.

Because SVIM, similar to other SV callers, analyzes read alignments it depends on the correctness of these alignments and inherits the limitations of the used read alignment method. One of these limitations originates from the repetitive nature of many genomes which keeps repetitive read segments from being mapped confidently. This can affect SVIM’s sensitivity but might also cause SVIM to classify an interspersed duplication as a novel insertion if the inserted segment cannot be uniquely mapped. This might particularly affect mobile element insertions whose individual copies are highly similar. Currently, SVIM is unable to detect chromosomal translocations and nested structural variants. We intend to add this functionality in the future. Additionally, we plan to implement genotyping capabilities for the detected variants in an upcoming release of SVIM.

## Supplementary Material

btz041_Supplementary_Materials
